# Structural insight into human N6amt1–Trm112 complex functioning as a protein methyltransferase

**DOI:** 10.1038/s41421-019-0121-y

**Published:** 2019-09-10

**Authors:** Wenjing Li, Yu Shi, Tianlong Zhang, Jie Ye, Jianping Ding

**Affiliations:** 1 State Key Laboratory of Molecular Biology, CAS Center for Excellence in Molecular Cell Science, Shanghai Institute of Biochemistry and Cell Biology, University of Chinese Academy of Sciences, Chinese Academy of Sciences, 320 Yue-Yang Road, Shanghai, 200031 China; 2 School of Life Science and Technology, ShanghaiTech University, 393 Hua-Xia Zhong Road, Shanghai, 201210 China

**Keywords:** X-ray crystallography, Post-translational modifications

## Abstract

DNA methylation is an important epigenetic modification in many organisms and can occur on cytosine or adenine. N^6^-methyladenine (6mA) exists widespreadly in bacterial genomes, which plays a vital role in the bacterial restriction-modification system. Recently, 6mA has also been reported to exist in the genomes of a variety of eukaryotes from unicellular organisms to metazoans. There were controversial reports on whether human N6amt1, which was originally reported as a glutamine MTase for eRF1, is a putative 6mA DNA MTase. We report here the crystal structure of human N6amt1–Trm112 in complex with cofactor SAM. Structural analysis shows that Trm112 binds to a hydrophobic surface of N6amt1 to stabilize its structure but does not directly contribute to substrate binding and catalysis. The active site and potential substrate-binding site of N6amt1 are dominantly negatively charged and thus are unsuitable for DNA binding. The biochemical data confirm that the complex cannot bind DNA and has no MTase activity for DNA, but exhibits activity for the methylation of Gln185 of eRF1. Our structural and biochemical data together demonstrate that N6amt1 is a bona fide protein MTase rather than a DNA MTase.

## Introduction

DNA methylation is an important modification which occurs mostly on C^5^-cytosine (5mC), N^4^-cytosine (4mC), and N^6^-adenine (6mA) in both prokaryotes and eukaryotes. Among these modifications, 6mA was initially discovered widespread in bacterial genomes and mainly functions as a part of the restriction-modification system to distinguish the host and foreign pathogenic DNAs in order to protect bacteria against viruses^1,2^. However, this modification was considered to be absent in metazoans. Recently, with the development of high-throughput sequencing technologies and highly sensitive MS approaches, 6mA was also reported to exist in the genomic DNAs of a variety of eukaryotes, including fungi, *Arabidopsis thaliana*, *Chlamydomonas reinhardtii*, *Caenorhabiditis elegans*, *Drosophila melanogaster*, zebrafish, mouse, pig, and human^3–11^. The abundance and distribution of 6mA on the genomes of these eukaryotes are quite different^12^. So far, limited functional studies indicate that DNA 6mA modification is a potential epigenetic mark and may play an important role in gene transcription and chromatin remodeling^12,13^.

With the discovery of 6mA on the genomic DNAs of multicellular eukaryotes, extensive efforts have been taken to identify the enzymes responsible for the writing and removal of this modification. N6amt1 (N^6^-adenine-specific DNA methyltransferase 1) was lately suggested to be the N^6^-adenine DNA methyltransferase (MTase) in human cells^10^. This study showed that overexpression of N6amt1 increases the DNA 6mA level, while knockdown of this gene decreases the 6mA modification level^10^. Besides, recombinant Flag-tagged N6amt1 exhibits 6mA modification activity towards DNA substrates^10^. In addition, in vitro overexpression or knockdown of N6amt1 in primary cortical neurons also results in increased or decreased 6mA level and accumulation of 6mA on the genome is correlated with extinction-induced gene expression^14^. However, there is also a contradicting report showing that deletion of N6amt1 in glioblastoma stem cells exhibits no effect on the 6mA level, and purified recombinant N6amt1 displays no detectable MTase activity towards DNA substrates in vitro^11^. Therefore, it is still controversial about whether N6amt1 is a bona fide N^6^-adenine DNA MTase in mammals.

N6amt1 was originally proposed as a SAM-dependent DNA MTase because it possesses the characteristic NPPY motif of bacterial N^6^-adenine and N^4^-cytosine DNA MTases^15,16^. Human N6amt1, also called HemK2, is distantly related to *E. coli* HemK (also called PrmC). Bacterial HemK was initially proposed to be a SAM-dependent DNA MTase which is directly involved in heme metabolism^17,18^. Later studies showed that *E. coli* HemK functions as a protein MTase which can methylate the polypeptide release factors RF1 and RF2^19,20^. Similarly, Mtq2p (also called YDR140w), the yeast homolog of HemK, was demonstrated to catalyze the methylation of the glutamine residue in the GGQ motif of eukaryotic release factor 1 (eRF1)^21^. Moreover, previous studies also showed that N6amt1 has no detectable DNA MTase activity^22^; instead, it forms a stable complex with a partner protein Trm112 and the complex functions as a glutamine-specific MTase for eRF1 in mammals^23^.

To investigate whether N6amt1 is a dual functional enzyme that could methylate both protein and DNA, we determined the crystal structure of human N6amt1–Trm112 complex bound with cofactor SAM and performed in vitro biochemical assays. Our structural and biochemical data together reveal that the active site and the putative substrate-binding site of N6amt1 are dominantly negatively charged that are unsuitable for DNA binding; the complex cannot bind DNA and has no DNA MTase activity; and instead, the complex exhibits an MTase activity for Gln185 of eRF1. These results demonstrate that N6amt1 is a protein MTase rather than a DNA MTase.

## Results

### Overall structure of human N6amt1–Trm112 complex

To investigate the structural basis for the function of human N6amt1, we expressed and purified human N6amt1–Trm112 complex from *E. coli*. Crystallization of the N6amt1–Trm112 complex in the absence and presence of cofactor *S*-adenosyl-methionine (SAM) both yielded crystals of the SAM-bound complex, indicating that SAM could be co-purified with the complex. The structure of the N6amt1–Trm112 complex was solved by the SAD method (Table 1). The crystal of the Se-Met derivative protein complex belongs to space group *I422* and contains two N6amt1–Trm112 molecules in the asymmetric unit. The crystal of the native protein complex belongs to space group *P6*_*1*_*22* and contains one N6amt1–Trm112 molecule in the asymmetric unit, and the structure of the native N6amt1–Trm112 complex was refined to 2.0 Å resolution (Table 1).

**Table 1:** Summary of diffraction data and refinement statistics

	Native	Se-Met
**Data collection**		
Wavelength (Å)	0.9778	0.9792
Space group	*P6*_*1*_ *22*	*I422*
Cell dimensions		
*a, b, c* (Å)	109.7, 109.7, 130.8	195.4, 195.4, 246.6
*α, β, γ* (°)	90, 90, 120	90, 90, 90
Resolution (Å)	50.0–2.00 (2.07–2.00)^a^	50.0–3.20 (3.31–3.20)
Observed reflections	206,280	1,141,273
Unique reflections (*I/σ(I)* > 0)	31,971 (3,126)	75,470 (7,531)
*R*_merge_ (%)^b^	11.5 (60.8)	22.0 (216.7)
*I*/σ (*I*)	16.7 (3.3)	17.3 (1.8)
Completeness (%)	99.7 (99.6)	100.0 (100.0)
Redundancy	6.5 (6.5)	15.1 (14.5)
**Refinement**		
Resolution (Å)	50.0–2.00	50.0–3.20
No. reflections		
Working set	31,895	39,509
Test set	1,664	3,775
R_work_/R_free_ (%)^c^	16.2/19.2	22.9/26.4
No. atoms		
Protein	2,498	5,041
Cofactor	27	54
Water	192	-
Wilson B-factors (Å^2^)	29.7	83.5
B-factors (Å^2^)		
Protein	37.3	78.8
Cofactor	26.5	71.0
Water	46.7	-
R.m.s. deviations		
Bond lengths (Å)	0.010	0.012
Bond angles (°)	1.1	1.3
Ramachandran plot (%)		
Favored	97.5	93.5
Allowed	2.5	6.5

^a^Numbers in parentheses represent the highest resolution shell

^b^R_merge_ = ∑_hkl_∑_i_|I_i_(hkl) −< I(hkl) > |/∑_hkl_∑_i_I_i_(hkl)

^c^R = ∑_hkl_||F_o_| − |F_c_||/∑_hkl_|F_o_|

**Fig. 1:**
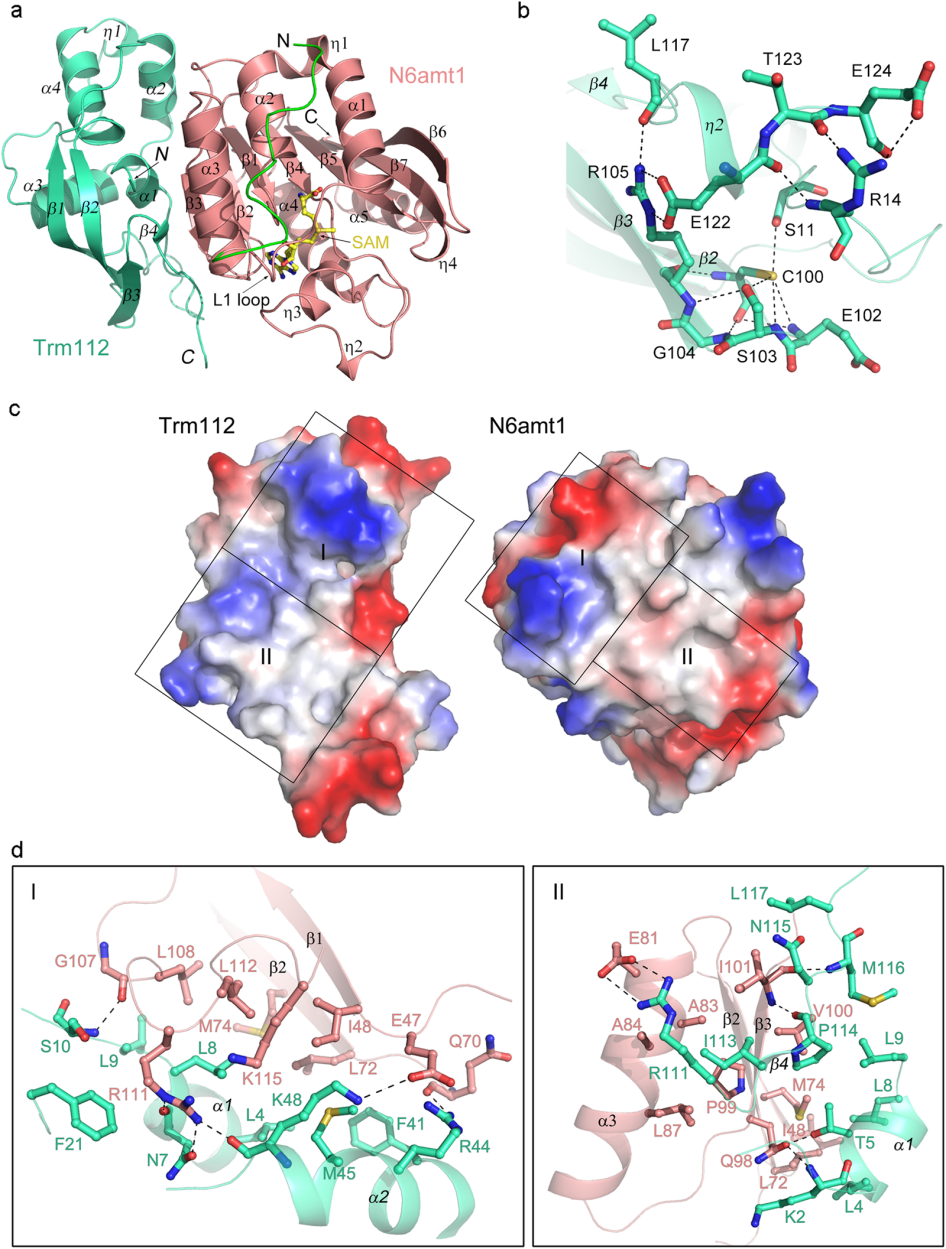
Crystal structure of human N6amt1–Trm112 complex. **a** Overall structure of Se-Met derivative N6amt1–Trm112 complex in ribbon diagram. N6amt1 and Trm112 are colored in salmon and green-cyan, respectively. The extra visible N-terminal loop of N6amt1 defined in the structure of the derivative complex is colored in green. The bound cofactor SAM is shown with a ball-and-stick model and colored in yellow. The secondary structure elements are indicated. The secondary structure elements of Trm112 are labeled in italics. **b** Structure of the “S(R/K)CS motif” of Trm112. The key residues involved in the hydrogen-bonding interactions with this motif are shown with ball-and-stick models. The hydrogen bonds are indicated with black dotted lines. **c** Electrostatic potential surfaces of Trm112 and N6amt1 at the interaction interface, which is the open-book view of Fig. 1a. The surface charge distribution is displayed as blue for positive, red for negative, and white for neutral. For clarity, the interface is divided into two regions to show the detailed interactions. **d** The zoom-in windows show the detailed interactions in the two regions of Fig. 1c. The residues involved in the interactions are shown with ball-and-stick models. The color coding is the same as Fig. 1a. The hydrogen bonds are indicated with black dotted lines

In the structure of the native N6amt1–Trm112 complex, all residues of Trm112 and most residues of N6amt1 except for the N-terminal residues 1–18 are clearly defined in the electron density. In the structure of the Se-Met derivative N6amt1–Trm112 complex, the N-terminal residues 5–18 of N6amt1 could be built with evident electron density despite a lower resolution of the structure (Fig. 1a and Supplementary Fig. S1). These results suggest that the N-terminal loop (L1 loop, residues 1–27) of N6amt1 is flexible in solution. Human N6amt1 adopts the typical fold of class I SAM-dependent MTases, which is composed of a central seven-stranded β-sheet sandwiched by three α-helices on one side (α1-α3) and two α-helices on the other (α4 and α5). Besides the consensus structural core of class I MTases, N6amt1 contains two auxiliary structure elements: a flexible L1 loop before the α1 helix and a small insertion domain between the β4 strand and the α4 helix (Fig. 1a). The insertion domain consists of three loops and two 3_10_ helices (η2 and η3). The active site is located near the C-terminus of the seven β-strands of the MTase domain and is partially covered by the insertion domain. Structural similarity search using the Dali server^24^ reveals that the structure of human N6amt1 shares the highest similarities with *Encephalitozoon cuniculi* (*E. cuniculi*) MTase Mtq2 and the MTase domain of *E. coli* HemK with a Z-score value of 24.2 and 22.0, respectively (Supplementary Table S1). Intriguingly, *E. cuniculi* Mtq2 methylates a Gln residue in the GGQ motif of eRF1^21^, and *E. coli* HemK carries out a similar reaction to modify RF1 and RF2^19,20^. This finding is consistent with previous studies showing that N6amt1 is a glutamine-specific MTase for eRF1 in mammals^23^.

The partner protein human Trm112 is composed of a helical domain formed by four α-helices (α1–α4) that packs against a twisted β-sheet formed by four anti-parallel β-strands (β1-β4) (Fig. 1a). The overall structure of human Trm112 is very similar to yeast and protozoan Trm112 in other MTase-Trm112 complexes, but resembles yeast Trm112 (an RMSD of 1.5 Å for 116 Cα atoms) more than protozoan Trm112 (an RMSD of 1.8 Å for 95 Cα atoms) (Supplementary Fig. S2a and Table S1). In all of the three reported MTase–Trm112 complexes, there is a Zn^2+^ bound to Trm112, which is located approximately at the end of the β2 strand. However, there is no metal ion bound with human Trm112 (Fig. 1b). In the yeast and protozoan Trm112, four Cys residues are involved in the binding of the metal ion, which also make extensive hydrogen-bonding interactions with the surrounding residues to further stabilize the zinc-binding domain formed by the N- and C-terminal extremities of the protein (Supplementary Fig. S2b). Nevertheless, in higher eukaryotes, three out of the four Cys residues are replaced with two Ser residues and one Arg or Lys residue (Supplementary Fig. S3a). In human Trm112, Ser11, Ser103, and Cys100 at the equivalent positions interact with each other by side chains and form hydrogen-bonding interactions with the surrounding residues (Glu102, Ser103, Gly104, and Arg105) via both main chains and side chains (Fig. 1b and Supplementary Fig. S3a). In addition, both the side chain and main chain of Arg14 form hydrophilic interactions with three conserved residues (Glu122, Thr123, and Glu124), which further stabilize the N- and C-terminal extremities of human Trm112. Thus, in higher eukaryotes, these four strictly or highly conserved residues constitute a non-zinc-binding motif “S(R/K)CS” of Trm112.

### Interactions between N6amt1 and Trm112

In the N6amt1–Trm112 complex, N6amt1 interacts with Trm112 mainly through a parallel β-zipper formed by the β3 strand of N6amt1 and the β4 strand of Trm112, yielding a continuous eleven-stranded β-sheet (Fig. 1a). Formation of the complex buries a total solvent-accessible surface area of 1200 Å^2^. The interaction interface exhibits both electrostatic and geometrical complementarities (Fig. 1c). Residues Ile48, Leu72, Met74, Ala83, Leu87, Pro99, Val100, Ile101, Leu108, and Leu112 of N6amt1 make hydrophobic interactions with residues Leu4, Leu8, Leu9, Phe41, Met45, Ile113, Pro114, and Leu117 of Trm112, which form a large hydrophobic region at the center of the interface (Fig. 1c, d). Apparently, formation of the complex shields the hydrophobic region of N6amt1 from exposure to the solvent. This explains the necessity to co-express N6amt1 with Trm112 to obtain a stable complex, similar to that of other MTase–Trm112 complexes^25–27^.

In addition, the hydrophobic core is surrounded by several hydrophilic interactions. Among them, the main-chain amine and carbonyl of Ile101 of N6amt1 form hydrogen bonds with the main-chain carbonyl of Pro114 and main-chain amine of Met116 of Trm112, respectively, which contribute to the formation of the parallel β-zipper at the interface (Fig. 1d, right panel). Besides, four residues of N6amt1 (Gln70, Gln98, Gly107, and Arg111) are engaged in seven hydrogen-bonding interactions with six residues of Trm112 (Lys2, Thr5, Asn7, Ser10, Arg44, and Lys48). Specifically, the main chain of Gln70 of N6amt1 forms a hydrogen bond with the side chain of Arg44 of Trm112; the side chain of Gln98 of N6amt1 forms two hydrogen bonds with the main-chain amide of Lys2 and the side chain of Thr5 of Trm112; the main-chain carbonyl of Gly107 of N6amt1 forms a hydrogen bond with the main-chain amide of Ser10 of Trm112; the side chain of Arg111 of N6amt1 forms three hydrogen bonds with the main-chain carbonyls of Asn7 and Lys48 and the side chain of Asn7 of Trm112. Moreover, the side chains of Glu47 and Glu81 of N6amt1 form salt bridges with the side chains of Lys48 and Arg111 of Trm112, respectively (Fig. 1d). Sequence alignment shows that most of the residues engaged in the formation of the complex are strictly or highly conserved in both proteins (Supplementary Fig. S3).

### Structures of the active site and the potential substrate-binding site

**Fig. 2:**
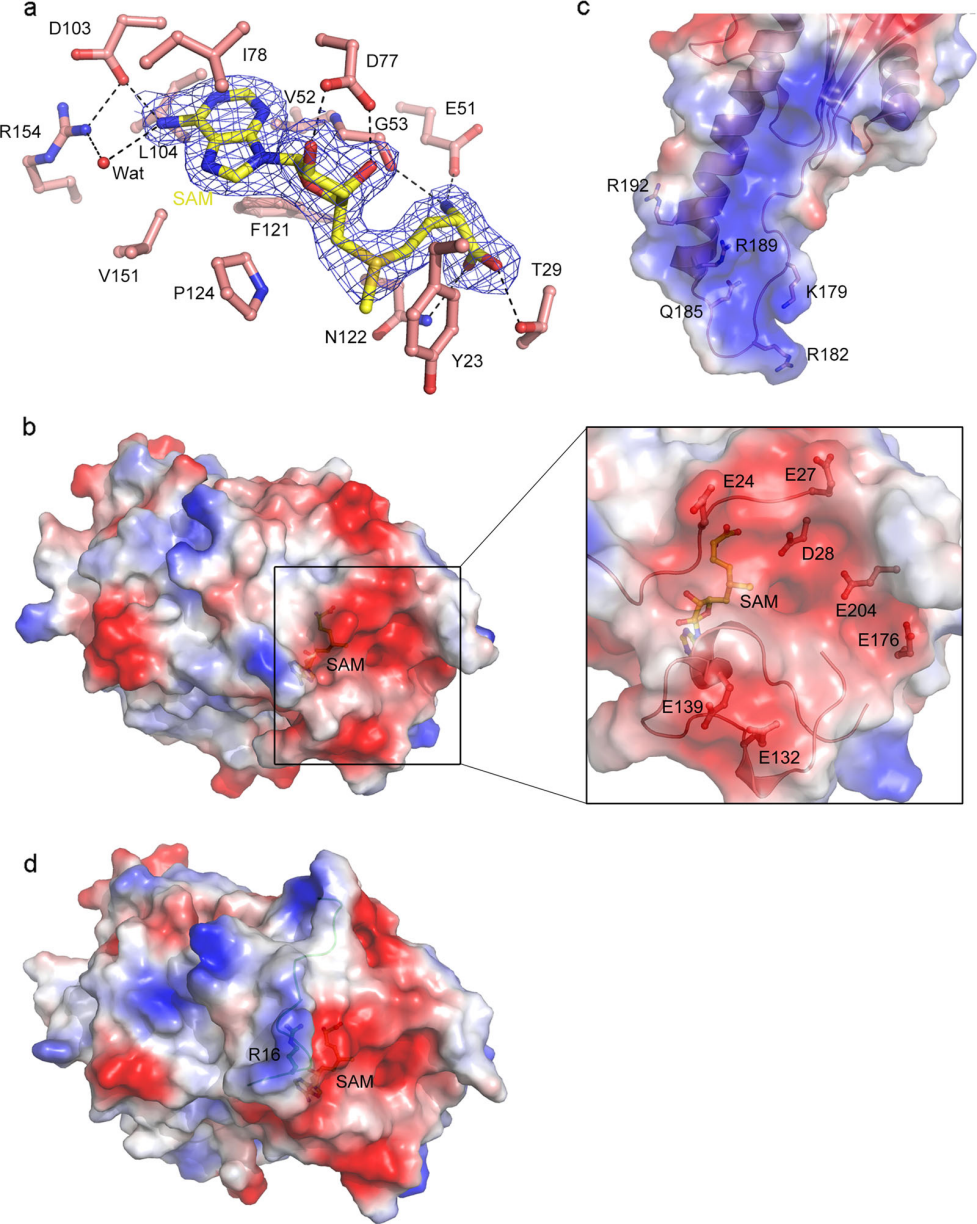
Structures of the active site and the potential substrate-binding site of N6amt1. **a** Interactions of SAM with N6amt1. The residues interacting with the cofactor are shown with ball-and-stick models. A water molecule involved in the hydrogen-bonding network is shown with a sphere and colored in red. A representative composite-simulated annealing omit map (*2Fo-Fc*, contoured at 1.0 σ level) for SAM is shown with the blue grids. **b** Electrostatic potential surface of native N6amt1–Trm112 complex in the same view as Fig. 1a. The zoom-in window shows the acidic residues composing the active site and the potential substrate-binding site. **c** Electrostatic potential surface around the GGQ motif of human eRF1 in the eRF1–eRF3 complex (PDB code 3E1Y). Gln185 and the adjacent basic residues are shown with ball-and-stick models. **d** Electrostatic potential surface of the Se-Met derivative N6amt1–Trm112 complex in the same view as Fig. 1a. The extra visible N-terminal loop is shown in ribbon and colored in green. The cofactor SAM and key residues are shown with ball-and-stick models

In the N6amt1–Trm112 complex, there is a SAM bound at the active site with well-defined electron density (Fig. 2a). The SAM binds to the C-terminal end of the central β-sheet of N6amt1 and assumes a bent conformation (Fig. 1a), similar to that in the structures of other class I SAM-dependent MTases^28^. The adenine moiety of SAM makes extensive hydrophobic interactions with the surrounding residues (Val52, Ile78, Leu104, Phe121, Pro124, and Val151), and additionally its N6 group forms a direct hydrogen bond with the side chain of Asp103 and an indirect hydrogen bond with the side chain of Arg154 via a water molecule (Fig. 2a). Both hydroxyl groups of the ribose form hydrogen bonds with the side chain of Asp77. The methionine moiety interacts with the side chain of Glu51 and the main-chain carbonyl of Gly53 via its amide group, and interacts with the side chains of Thr29 and Asn122 via its carboxyl group. The side chain of Tyr23 packs beside the methyl group and the sulfur atom of SAM and may play a role in the process of methylation. Sequence alignment shows that the key residues involved in the SAM binding are strictly or highly conserved in N6amt1 from different species (Supplementary Fig. S3b).

Since it is still a controversial issue about whether N6amt1 is a bona fide 6mA DNA MTase in mammals, we performed structural analysis to explore the substrate specificity of N6amt1. Structural similarity search shows that N6amt1 shares the highest similarities with Mtq2 and HemK, and resembles Mtq2 more than HemK (Supplementary Table S1 and Fig. S4a). The previous structural and functional studies have revealed that most of the class I MTases identified so far contain accessory structure elements that are inserted throughout the consensus seven-β-stranded fold and seem to facilitate substrate binding and recognition^28^. Besides the MTase domain, *E. coli* Hemk contains a large N-terminal domain which is involved in the recognition and binding of the substrate RF1 (Supplementary Fig. S4a). However, the accessory structure elements of N6amt1 and Mtq2 are relatively small (Fig. 1a and Supplementary Fig. S3b). Similar to Mtq2, N6amt1 exhibits a relatively flat, open, and negatively charged surface surrounding the methyl group of SAM, which is presumably the substrate-binding site (Fig. 2b and Supplementary Fig. S4b). This negatively charged surface is composed of several acidic residues, including Glu24 and Glu27 from the L1 loop, Glu132 and Glu139 from the insertion domain, and Asp28, Glu176, and Glu204 from the MTase domain, which could contribute to the substrate binding and recognition (Fig. 2b). It is obvious that this negatively charged substrate-binding site of N6amt1 is unsuitable for the binding of negatively charged phosphate backbone of a DNA substrate. On the other hand, structural analysis of the reported human eRF1–eRF3 complex shows that Gln185 of human eRF1 is surrounded by several basic residues and particularly Lys179, Arg182, Arg189, and Arg192 form a positively charged surface patch, suggesting that eRF1 is a suitable substrate for N6amt1 (Fig. 2c).

In the structure of the native N6amt1–Trm112 complex, the N-terminus of the L1 loop is disordered and the L1 loop contains only residues 19–27; however, in the structure of the Se-Met derivative N6amt1-Trm112 complex, residues 5–18 of the L1 loop were defined (Fig. 1a). The extra visible region of the L1 loop consists of three basic residues (His11, His13, and Arg16) which form a small positively charged surface patch adjacent to but does not alter the overall charge properties of the potential substrate-binding site (Fig. 2d). Considering that the L1 loop of N6amt1 is relative flexible in solution, whether it would contribute to the binding and recognition of a DNA substrate will be verified by biochemical studies later.

In the N6amt1–Trm112 complex, a largely hydrophobic surface of N6amt1 is shielded by Trm112 from exposure to the solvent (Fig. 1c). N6amt1 could efficiently methylate Gln185 of human eRF1 only when it forms a complex with Trm112^23^. In addition to N6amt1 and its yeast homolog Mtq2, Trm112 can also form complexes with other class I MTases, including Bud23, Trm9, and Trm11, which catalyze the methylation of different substrates including rRNA and tRNA^25–27,29^. In those MTase–Trm112 complexes, Trm112 was suggested to function as a partner and activator of the MTases^30^. To investigate whether Trm112 plays a role in the substrate binding and recognition of N6amt1, we compared the structure of the N6amt1-Trm112 complex with the reported structures of the Bud23-Trm112 and Trm9-Trm112 complexes^25,26^. Structural comparison shows that Bud23 and Trm9 can be superimposed well with N6amt1 despite a low sequence identity (Supplementary Table S1 and Fig. S4a). Moreover, in all of the MTase-Trm112 complexes, Trm112 interacts with the MTases in a similar manner (Supplementary Fig. S4a). In particular, the β4 strand of Trm112 interacts with the β3 strand of the MTases to form a parallel β-zipper, yielding a continuous eleven-stranded β-sheet. In addition, several hydrophilic interactions between N6amt1 and Trm112 could also be found in the other MTase-Trm112 complexes. For example, the hydrophilic interactions between Gln98 of N6amt1 and Lys2 and Thr5 of Trm112 and between Arg111 of N6amt1 and Asn7 and Lys48 of Trm112 have corresponding interactions in the Bud23–Trm112 and Trm9–Trm112 complexes (Supplementary Fig. S4c). These results indicate that the interaction mode of Trm112 with those MTases is conserved from yeast to human. In all of the three MTases, the active site is located near the C-terminus of the seven β-strands of the MTase domain. Nevertheless, the structure surface surrounding the active site in Bud23 and Trm9 is largely positively charged, which is suitable for the binding of an RNA substrate; in contrast, the structure surface surrounding the active site in N6amt1 is largely negatively charged, which is unsuitable for the binding of a nucleotide substrate (Supplementary Fig. S4b and Fig. 2b). As Trm112 binds distantly from the active site of the MTase in those complexes, its binding appears to have no direct impacts on the chemical properties and structures of the active site and the substrate-binding site, suggesting that Trm112 is not directly involved in the substrate binding and recognition. However, the previous biochemical studies of the Mtq2–Trm112 and Trm11–Trm112 complexes showed that mutations of several residues located on the surface of Trm112 away from the interaction interface with the MTases could affect the enzymatic activities of MTases but have no effects on the complex formation or the SAM binding^27,29^. Thus, we cannot exclude the possibility that in addition to having a role in the stabilization of MTases, Trm112 might also play a role in the substrate binding and activity modulation of MTases via allosteric regulation^30^.

### Biochemical and mutagenesis analyses

**Fig. 3:**
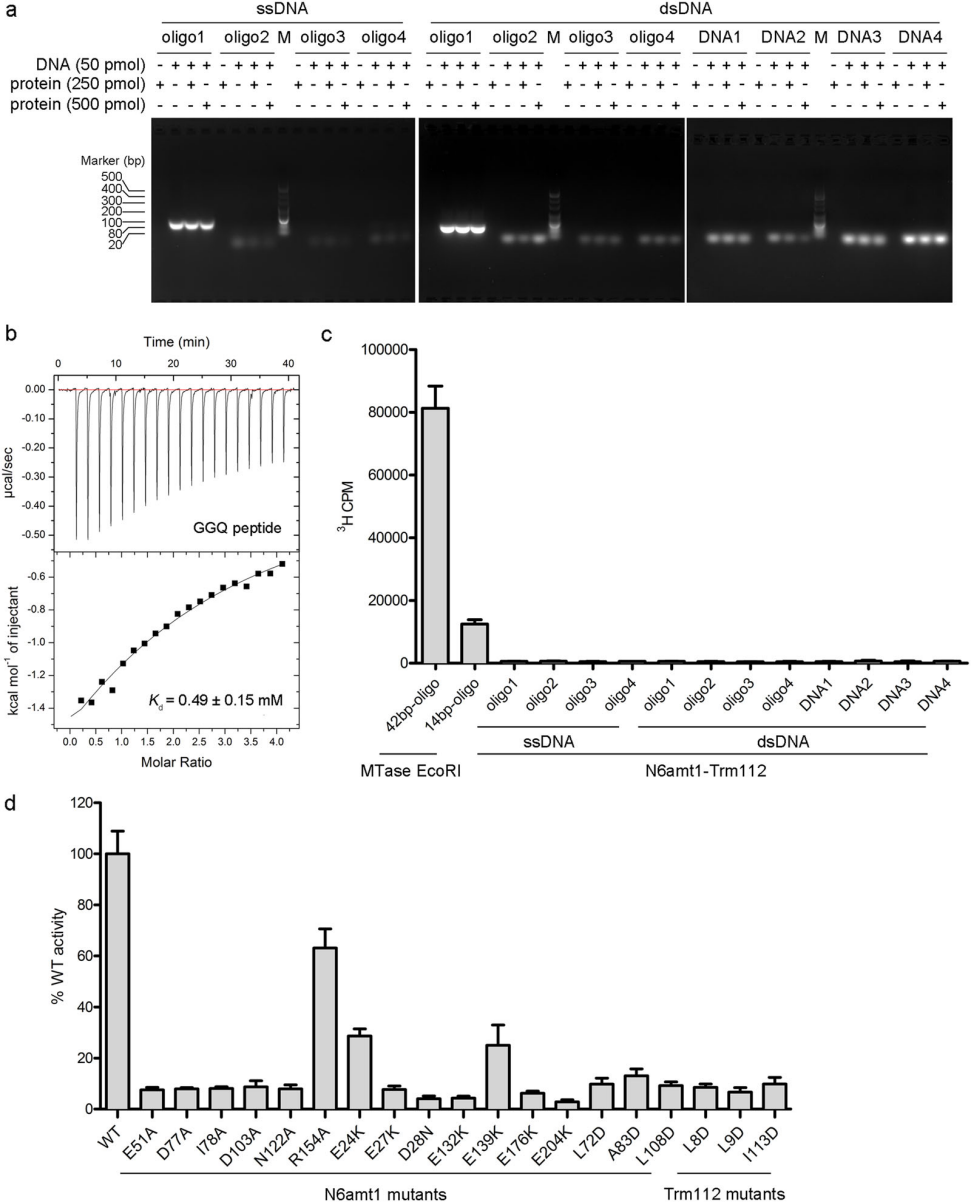
Biochemical studies of the N6amt1–Trm112 complex. **a** EMSA analysis of N6amt1–Trm112 towards different ssDNA and dsDNA. Oligos1–4 are the four DNA oligos used by Xiao *et al*. in the examination of the DNA 6mA modification activity of N6amt1^10^. The other four oligos are as follows: DNA1, CTTTTTAGAAGCACA; DNA2, AATGAGAAGTTTATC; DNA3, AAAATCGAGGTTCCC; DNA4, ACTGACCATGGATCG. **b** ITC analysis of the binding affinity of N6amt1-Trm112 towards the 15-residue GGQ peptide of eRF1. Prior to titration, the protein complex was incubated with SAH. The dissociation constant (*K*_d_) is indicated. **c** Enzymatic activity assay of N6amt1–Trm112 towards dsDNAs and ssDNAs with different lengths and sequences. MTase EcoRI is used as a positive control. All the experiments were performed in triplicates and the error bars represent the standard error of the mean. **d** Effects of mutations of the key residues at the active site of N6amt1 and the N6amt1–Trm112 interface on the enzymatic activity of N6amt1-Trm112 for the methylation of human eRF1. The average of ^3^H incorporation measurement of the wild-type protein complex (about 2300 c.p.m.) is defined as “100% WT activity”, and the MTase activities of the N6amt1–Trm112 mutants are shown relative to 100% WT activity. All the experiments were performed in triplicates and the error bars represent the standard error of the mean

Previous studies on DNA 6mA modification in eukaryotes showed that the abundance and distribution of 6mA on the genomes and the identified 6mA enriched sequence motifs vary substantially in different eukaryotes. For instance, 6mA is enriched at the transcription start sites and an ApT dinucleotide motif was suggested to be the consensus sequence in *C. reinhardtii*^3^. However, 6mA is broadly and evenly distributed on the genome and two sequence motifs (AGAA and GAGG) were reported to associate with the presence of 6mA modification in *C. elegans*^4^. Furthermore, 6mA is shown to be highly enriched in exon regions in human genome and the [G/C]AGG[C/T] motif was identified to be significantly associated with 6mA modification^10^. Thus, to investigate whether the N6amt1–Trm112 complex could bind DNA, we first synthesized four DNA oligos according to those reported 6mA-enriched sequence motifs and four other DNA oligos used by Xiao et al. in the examination of the DNA 6mA modification activity of N6amt1^10^, and performed in vitro electrophoretic mobility shift assay (EMSA). Our results show that N6amt1–Trm112 has no detectable binding with the tested dsDNAs or ssDNAs with different lengths and sequences, indicating that N6amt1–Trm112 cannot bind DNA in vitro (Fig. 3a).

As N6amt1 was reported to function as a glutamine-specific MTase for eRF1^23^ and could catalyze the methylation of a Gln185-centered 15-residue peptide of human eRF1 with the sequence of KKHGRGGQSALRFAR (hereafter named as GGQ peptide)^31^, we then carried out isothermal titration calorimetry (ITC) experiment to examine the binding affinity between N6amt1–Trm112 and the GGQ peptide. Our result shows that the protein complex possesses the ability to bind the GGQ peptide albeit with a relatively low binding affinity (0.49 ± 0.15 mM) (Fig. 3b). These results are consistent with the structural data showing that the potential substrate-binding site of N6amt1 is largely negatively charged, which is suitable for the binding of basic residue-enriched substrate peptide but unsuitable for the binding of DNA. Taken the structural and biochemical data together, the N6amt1–Trm112 complex exhibits binding affinity towards the GGQ peptide of eRF1 but not dsDNA or ssDNA.

We further performed in vitro MTase activity assays to explore the substrate specificity of N6amt1. Consistent with the in vitro binding assay results, our activity assay results show that the N6amt1–Trm112 complex could not catalyze the methylation of dsDNA or ssDNA oligos with different lengths and sequences (Fig. 3c). As a positive control, the MTase EcoRI could catalyze the methylation of DNA substrates with high efficiency (Fig. 3c). On the other hand, the N6amt1–Trm112 complex could methylate the protein substrate eRF1 effectively (Fig. 3d).

To verify the functional roles of the key residues at the active site of N6amt1 and the N6amt1–Trm112 interface in the catalytic reaction, we performed mutagenesis and biochemical studies (Fig. 3d). Single mutation of any of the residues involved in the cofactor binding (Glu51, Asp77, Ile78, Asp103, and Asn122) of N6amt1 results in almost complete loss of the activity towards eRF1 as these mutations would affect the binding of SAM. Mutation R154A leads to about 40% loss of the activity towards eRF1, suggesting that Arg154 plays a minor role in the cofactor binding. Mutations of most of the acidic residues (Glu27, Asp28, Glu132, Glu176, and Glu204) at the potential substrate-binding site of N6amt1 also abolish the activity towards eRF1, indicating that these residues play important roles in the substrate binding. Besides, mutations of Glu24 and Glu139 at the potential substrate-binding site of N6amt1 moderately decrease the activity of N6amt1, suggesting that these two residues participate in but not function as key residues in the substrate binding. In addition, single mutations of residues at the N6amt1–Trm112 interface (Leu72, Ala83, and Leu108 of N6amt1; Leu8, Leu9, and Ile113 of Trm112) severely impair the activity towards eRF1, indicating that formation of the protein complex is vital for its activity. Taken together, our structural and biochemical data demonstrate that N6amt1–Trm112 is able to bind and catalyze the methylation of eRF1 but not DNA, and thus is a bona fide protein MTase rather than a DNA MTase. In addition, the partner protein Trm112 is essential for the stability of N6amt1 and might play an indirect role in the substrate binding and activity modulation of N6amt1 via allosteric regulation.

## Discussion

With the discovery of 6mA modification in the genomic DNAs of some eukaryotes, lots of efforts have been put into looking for the protein machineries that are responsible for the establishment and removal of this modification. The specific demethylases of DNA 6mA modification have been reported and verified by several groups^4,5,11^; however, the specific DNA MTase(s) remain unknown. Whether N6amt1 is the bona fide N^6^-adenine DNA MTase in mammals is still in debate. In this work, we carried out the structural study of human N6amt1–Trm112 complex and performed in vitro biochemical studies to investigate the substrate specificity of N6amt1.

*E. coli* HemK, which is distantly related to human N6amt1, was shown to catalyze the methylation of the polypeptide release factors RF1 and RF2^19,20^. N6amt1 was also reported to form a heterodimer with Trm112 and functions as a glutamine-specific MTase for eRF1 in mammals^23^. Further studies revealed that N6amt1 could methylate additional protein substrates besides eRF1 and its methylation activity requires a GQX_3_R motif^31^, suggesting that the substrate specificity of N6amt1 is relatively low. Although N6amt1 is distantly related to *E. coli* HemK, the structural characteristics of these enzymes beyond the MTase domain is quite different. Besides the MTase domain, *E. coli* HemK contains a large N-terminal domain which interacts extensively with the RF1 substrate (Supplementary Fig. S4a)^32^. In addition, the substrate-binding site of *E. coli* HemK is relatively closed and the Gln residue of the substrate is inserted into a narrow channel at the active site (Supplementary Fig. S4b). These results suggest that *E. coli* HemK might have a relatively high substrate specificity. In contrast, our structural study of the N6amt1–Trm112 complex shows that the potential substrate-binding site of N6amt1 is relatively flat and open, and additionally the accessory structure elements beyond the MTase domain are relatively small in size and are composed of loops which may undergo conformational changes to bind different substrates (Figs. 1a and 2d). These results are in agreement with the functional data that N6amt1 has a low substrate specificity.

**Fig. 4:**
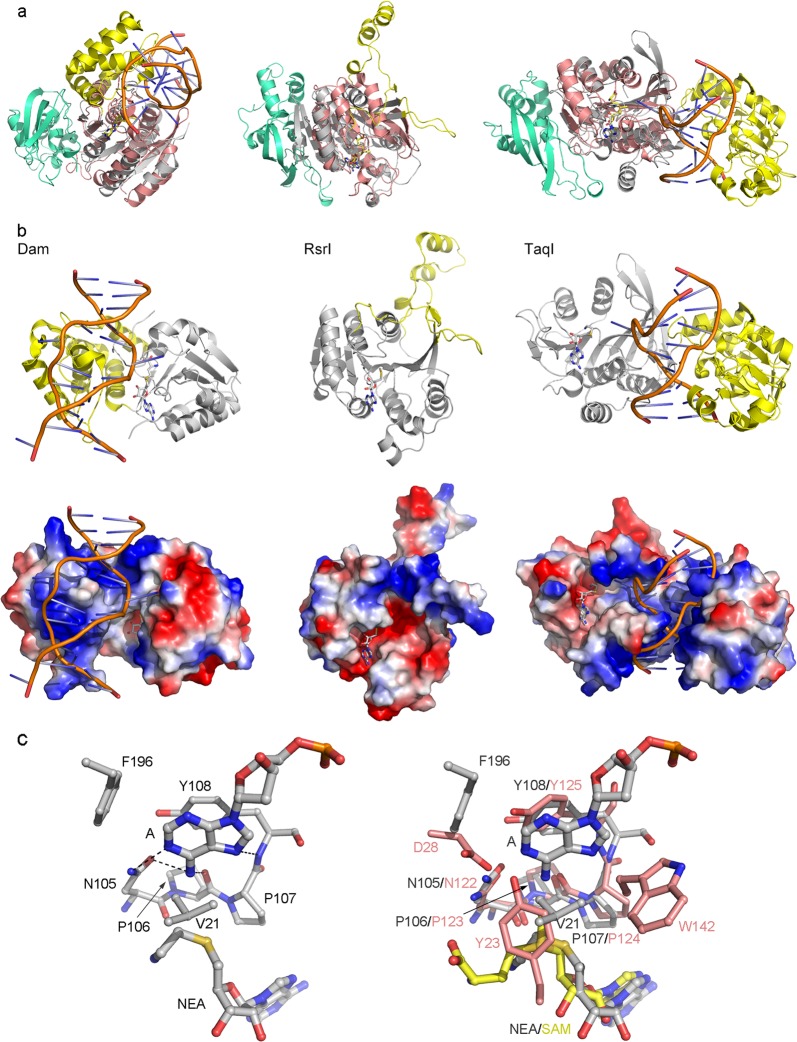
Structural comparisons of human N6amt1–Trm112 complex with representative bacterial DNA 6mA MTases. **a** Structural comparisons of N6amt1-Trm112 with Dam (left panel, PDB code 2G1P), RsrI (middle panel, PDB code 1EG2), and TaqI (right panel, PDB code 1G38). **b** Overall structures and Electrostatic potential surfaces of Dam, RsrI, and TaqI. The MTase and TRD domains of the bacterial DNA 6mA MTases are shown with ribbon models and colored in gray and yellow, respectively. The bound dsDNA is shown with orange ribbon. **c** Structure of the active site of TaqI (left panel) and comparison of the active site of N6amt1 with that of TaqI (right panel)

Nowadays, there were contradicting reports about whether N6amt1 is a bona fide N^6^-adenine DNA MTase in mammals. N6amt1 was originally proposed as a SAM-dependent DNA MTase because it possesses the characteristic NPPY motif (motif IV) of bacterial N^6^-adenine and N^4^-cytosine DNA MTases^15,16^. DNA MTases include C5 MTases responsible for C^5^-cytosine methylation and amino MTases responsible for N^6^-adenine and N^4^-cytosine methylations. Among them, all the C5 MTases consist of ten consecutive conserved motifs (I–X), as well as a target recognition domain (TRD) near the C-terminus. Similar to the C5 MTases, the amino MTases possess one TRD but only nine of the ten conserved motifs (I–VIII and X). The amino MTases could be further divided into three groups (α, β, and γ) according to the orders of the nine conserved motifs, and all the three groups contain DNA 6mA MTases^15,33^. So far, the crystal structures of several bacterial DNA 6mA MTases have been reported. Thus, we performed structural comparisons of the N6amt1–Trm112 complex with the DNA 6mA MTases Dam, RsrI, and TaqI as the representatives of the α, β, and γ groups of amino MTases, respectively. Like N6amt1, all the three bacterial DNA 6mA MTases belong to the class I MTase family^28^. The MTase domain of these enzymes can be superimposed well with that of N6amt1 (an RMSD of 3.0 Å for 140 Cα with Dam; an RMSD of 3.3 Å for 50 Cα with RsrI; an RMSD of 2.2 Å for 136 Cα with TaqI) (Fig. 4a). The structural comparison shows that the TRD of Dam, RsrI, and TaqI is located adjacently to the MTase domain and constitutes parts of the largely positively charged surface around the active site to facilitate the binding of DNA substrate (Fig. 4b). Structural analysis shows that the number of residues constituting the core MTase domain of the class I MTases is about 150 amino acids on average^28^. The full-length N6amt1 contains only 214 amino acids and the accessory structure elements (the L1 loop and the insertion domain) comprise of only about 50 residues (Fig. 1a and Supplementary Fig. S3b). The relatively small and mainly negatively charged accessory structure elements of N6amt1 are apparently unfavorable for the binding of acidic DNA substrate to its active site (Fig. 2).

Structure analysis of the bacterial DNA 6mA MTases reveals that motifs X, I, II, and III constitute the SAM-binding region and motifs IV–VIII constitute the catalytic region. In all the three groups of amino MTases, the N-terminus of groups α and γ begins with the SAM-binding region, while that of group β starts with the catalytic region^15^. Analyses of the sequence position and structural location of the NPPY motif of N6amt1 show that this enzyme resembles groups α and γ amino MTases more than group β amino MTases. As the adenine base to be methylated was flipped out of the duplex DNA and inserted into the active site of TaqI in the TaqI–DNA structure, we compared the active site of N6amt1 with that of TaqI. The comparison shows that the NPPY motifs of N6amt1 and TaqI could be aligned well despite low sequence and structural similarities of the surrounding residues (Fig. 4c). In the TaqI-DNA structure, the nucleophilic nitrogen of adenine forms two hydrogen bonds with the side-chain carbonyl of Asn105 and the main-chain carbonyl of Pro106, and the purine ring is stabilized via a π-π stacking interaction with the side chain of Tyr108 (Fig. 4c). In the structure of the HemK-RF1 complex, the glutamine residue of RF1 is inserted into the active site of HemK and stabilized by the NPPY motif in a similar way as the DNA MTase TaqI (Supplementary Fig. S4d). The proposed catalytic mechanism suggests that the hydrogen-bonding interaction between the amino group of the substrate and the NPPY motif would polarize and activate the amino group for acceptance of the methyl group from SAM^28,34^. This suggests that the NPPY motif at the active site of N6amt1 might have the ability to catalyze the methylation of adenine in DNA.

Several previous studies showed that N6amt1 has no detectable DNA MTase activity^22,23^, and could not be a DNA 6mA MTase in glioblastoma stem cells^11^. Consistently, our structural and biochemical data demonstrate that the N6amt1–Trm112 complex does not possess the abilities to bind DNA and to catalyze the methylation of DNA substrate. Nonetheless, a previous study showed that the recombinant Flag-tagged N6amt1 exhibits 6mA modification activity towards four synthetic DNA oligos^10^. As the Flag-tagged N6amt1 was expressed and purified from mammalian cells, it is possible that the protein sample may contain some endogenous DNA MTase(s) that are responsible for the DNA 6mA modification. On the other hand, as discussed above, despite the acidic property of the putative substrate-binding site of N6amt1, the chemistry of the active site of N6amt1 has the potential to catalyze the methylation of base moiety of nucleotides. As N6amt1 is a relatively small MTase with small accessory structure elements and its partner protein Trm112 is involved in the stabilization of N6amt1 but not directly involved in the substrate binding, we cannot rule out the possibility that there might be additional partner protein(s) which can interact with N6amt1 to cover up the negatively charged surface surrounding the active site and exert the function of the TRD of bacterial DNA MTases to facilitate the binding of DNA substrates. In this way, N6amt1 may act as a dual functional MTase that could catalyze the methylation of both protein and DNA and its activity could be regulated by different partner proteins.

During the preparation of our manuscript, another group published a study on the structure and function of the N6amt1–Trm112 complex^35^. In their work, the authors found that the protein complex could catalyze the mono-methylation of histone H4K12, and solved the crystal structure of human N6amt1–Trm112 in complex with SAH and an H4K12me1 peptide. In the structure, the H4K12 peptide is bound to the acidic surface patch of N6amt1 which is identified as the potential substrate-binding site in this work^35^. Their findings are in agreement with our results that the dominantly negatively charged substrate-binding site of N6amt1 is unsuitable for DNA binding. These results together indicate that N6amt1 functions as a protein MTase that could methylate histone H4, eRF1, and additional protein substrates^31,35^.

To date, the available structural and biochemical data have demonstrated that N6amt1 possesses MTase activity towards both eRF1 and H4. In the structure of N6amt1–Trm112 in complex with an H4K12me1 peptide, the ε-amine atom of the methylated lysine of the H4K12 peptide forms three hydrogen bonds with the side-chain carbonyls of Asp28 and Asn122 and the main-chain carbonyl of Pro124 (Supplementary Fig. S4e). As there is no structure of N6amt1–Trm112 in complex with eRF1 available, we docked a glutamine residue into the active site of N6amt1 by superposing *E. coli* HemK2–RF1 complex with N6amt1–Trm112, which shows that the side-chain amino group of the glutamine residue of RF1 is located in adjacent to that of the methylated lysine residue of H4K12, suggesting that the glutamine residue of eRF1 might be recognize by N6amt1 in a similar manner as the lysine residue of H4K12 (Supplementary Fig. S4e). In addition, structural comparison of the two N6amt1–Trm112 complexes shows that the key residues forming the active site of N6amt1 undergo no major conformational changes upon the substrate binding (Supplementary Fig. S4e). These results indicate that the active site of N6amt1 can accommodate and methylate both lysine of H4K12 and glutamine of eRF1.

## Materials and methods

### Cloning, expression, and purification

The genes encoding the full-length human N6amt1, Trm112, and eRF1 were amplified by PCR from cDNAs of 293 T cells. For expression of the N6amt1–Trm112 complex, the *N6amt1* and *Trm112* genes were cloned into the MCS1 and MCS2 sites of the pET-Duet vector (Novagen), respectively, which adds a His_6_ tag at the N-terminus of N6amt1. For expression of eRF1, the *eRF1* gene was cloned into the V28E vector (kind gift from Dr. Tengchuan Jin, School of Life Sciences, University of Science and Technology of China)^36^, which attaches a MBP tag and a His_6_ tag at the N-terminus and C-terminus of eRF1, respectively. The constructed plasmids were transformed into *E. coli* BL21(DE3) Condon Plus strain. The transformed bacterial cells were grown in LB medium containing 0.1 mg/ml ampicillin at 37 °C to OD_600_ of 0.6, and then induced with 0.2 mM IPTG at 16 °C overnight. The cells were collected, resuspended, and lysed on ice by high-pressure crushing in buffer A (20 mM Tris–HCl, pH 7.5, and 100 mM NaCl) supplemented with 2 mM β-mercaptoethanol and 1 mM phenylmethylsulfonyl fluoride. The cell debris was precipitated by centrifugation and the supernatant was collected for protein purification.

The N6amt1–Trm112 complex was purified by affinity chromatography using a Ni-NTA column (Qiagen) with buffer A supplemented with 20 mM imidazole and 200 mM imidazole serving as the washing buffer and elution buffer, respectively. The proteins were further purified with gel filtration using a Superdex G200 16/600 column (GE Healthcare) pre-equilibrated with buffer B (20 mM Tris–HCl, pH 7.5, 100 mM NaCl, and 1 mM dithiothreitol). Purification of the MBP-eRF1-His_6_ protein followed the same procedures except that no dithiothreitol was added in buffer B in gel filtration. Expression and purification of the Se-Met substituted N6amt1–Trm112 complex followed the same procedures as the native protein complex except that the bacterial cells were grown in M9 medium. Constructs of the N6amt1 and Trm112 mutants containing point mutations were generated using the QuikChange Site-Directed Mutagenisis kit (Stratagene) and verified by sequencing. Expression and purification of the mutants were the same as the wild-type proteins. The purified proteins were of high purity (above 95%) as analyzed by SDS–PAGE.

### Crystallization, data collection, and structure determination

The purified N6amt1–Trm112 complex was concentrated to about 16 mg/ml in buffer B. Crystallization was carried out using the hanging drop vapor diffusion method at 16 °C by mixing equal volume of protein solution and reservoir solution. Crystals of the native protein complex were grown in drops containing the reservoir solution (0.1 M succinic acid, pH 7.0, and 15% PEG3350). Crystals of the Se-Met derivative protein complex were grown in drops containing the reservoir solution [1.6 M (NH_4_)_2_SO_4_, 0.1 M MES, pH 6.5, and 10% 1,4-Dioxane]. The native crystals were cryoprotected using oil and the Se-Met derivative crystals were cryoprotected using the reservoir solution supplemented with 30% glycerol, and then flash-cooled into liquid N_2_. Diffraction data were collected at 100 K at BL17U1 of Shanghai Synchrotron Radiation Facility and BL19U1 of National Facility for Protein Science Shanghai, and processed with HKL2000^37^. The statistics of the diffraction data are summarized in Table 1.

The structure of the Se-Met derivative N6amt1–Trm112 complex was solved using the single-wavelength anomalous dispersion (SAD) method as implemented in Phenix^38^. The structure of the native N6amt1–Trm112 complex was solved by the molecular replacement (MR) method using the derivative complex structure as the search model. Model building was performed with Coot^39^ and structure refinement was carried out using Phenix^38^ and Refmac5^40^. Structural analysis was carried out using programs in CCP4^41^ and the PISA server^42^. The structure figures were generated using Pymol (www.pymol.org)^43^. The statistics of the structure refinement and the quality of the final structure models are also summarized in Table 1.

### In vitro MTase activity assays for eRF1 and DNA

The MTase activity assay of N6amt1 for eRF1 was carried out at 37 °C in a reaction mixture of 50 μl consisting of 4 pmol N6amt1-Trm112, 1μl ^3^H-labeled SAM (PerkinElmer) as the methyl donor, and 40 pmol recombinant MBP-eRF1 protein as the substrate in the reaction buffer (10 mM Tris–HCl, pH 7.6, 50 mM KCl, 10 mM MgAc_2_, and 14 mM β-mercaptoethanol). After 1 h, the reaction mixture was loaded onto a Waterman P81 filter paper, which was air dried and then washed with 10% trichloroacetic acid (TCA) thrice and with acetone for 10 min. The MTase activity assay of N6amt1 for DNA was carried out at 25 °C overnight in a reaction mixture of 50μl consisting of 20 pmol N6amt1–Trm112, 2.5μl ^3^H-labeled SAM, and 250 pmol DNA oligo as the substrate in the reaction buffer. The reaction mixture was loaded onto a Waterman 3 filter paper, which was air dried and then washed with 10% TCA thrice and with ethanol for 20 min. The amount of radioactivity incorporated into the protein or DNA substrate was quantified with liquid scintillation. All the experiments were performed in triplicates and the error bars represent the standard error of the mean.

### Isothermal titration calorimetry analysis

The binding thermodynamic parameters between N6amt1–Trm112 and a 15-residue peptide of eRF1 centered at Gln185 (KKHGRGGQSALRFAR) were measured using the isothermal titration calorimetry (ITC) method with an ITC200 Micro-calorimeter (MicroCal) at 20 °C. Prior to titration, the protein complex was incubated with *S*-adenosyl-homocysteine (SAH) at the molar ratio of 1:2. In the experiment, the initial injection of 0.4μl of the peptide solution was discarded to eliminate the effect of titrant diffusion across the syringe tip during the equilibration process, and the dataset consisted of 20 injections of 2μl of 2 mM peptide into the sample cell containing 250μl of 0.1 mM N6amt1–Trm112. The heats of dilution were negligible in the experiment. The binding constants and other thermodynamic parameters were determined by fitting the integrated titration data using a single binding site model by a nonlinear least-squares method implemented in MicroCal Origin software version 7.0.

### Electrophoretic mobility shift assay

EMSA was performed to investigate whether N6amt1–Trm112 possesses DNA-binding ability with several dsDNA and ssDNA oligos of different lengths and sequences. Specifically, 20μl of reaction mixture consisted of 50 pmol DNA in a binding buffer containing 10 mM Tris-HCl (pH 7.5), 50 mM KCl, and 10 mM MgAc_2_. Reaction was initiated by adding different amounts of N6amt1–Trm112 (250 or 500 pmol) and then incubated at 25 °C for 2 h. The reaction mixture was loaded on a 2.5% agarose gel and visualized under UV with Gel Green (Biotium) staining.

### Accession codes

The crystal structures of the native and Se-Met derivative N6amt1–Trm112 complexes have been deposited in the Protein Data Bank under accession codes 6KMR and 6KMS, respectively.

## Supplementary information

Supplementary Information.

## Supplementary information

**Supplementary Information** accompanies the paper at (10.1038/s41421-019-0121-y).
