# Supplementary material for: Structural insight into human N6amt1–Trm112 complex functioning as a protein methyltransferase
Source: Cell Discov. 2019 Sep 10;5:51. doi: 10.1038/s41421-019-0121-y (PMC6796863; doi:10.1038/s41421-019-0121-y)
Supplement: Supplementary file 1 — Supplementary Information. [file 41421_2019_121_MOESM1_ESM.docx]

Supplementary Information

**Supplementary Table S1.** Structural similarities of human N6amt1 (HsN6amt1) and human Trm112 (HsTrm112) with representative MTases and Trm112 from other species, respectively. The abbreviations are as follows: EcMtq2-Trm112, *Encephalitozoon cuniculi* Mtq2-Trm112; YlTrm9-Trm112, *Yarrowia lipolytica* Trm9-Trm112; ScBud23-Trm112, *Saccharomyces cerevisiae* Bud23-Trm112; EcHemK, *Escherichia coli* HemK.

|  |  | **EcMtq2-Trm112** | **YlTrm9-Trm112** | **ScBud23-Trm112** | **EcHemK** |
| --- | --- | --- | --- | --- | --- |
|  | PDB code | 3Q87 | 5CM2 | 4QTT | 2B3T |
| **HsN6amt1** | Identity (%) | 37.9 | 9.7 | 11.9 | 20.0 |
|  | RMSD (Å) | 1.4 | 3.3 | 3.2 | 1.9 |
|  | Aligned Cα | 161 | 113 | 135 | 170 |
|  | Z-score | 24.2 | 13.2 | 14.7 | 22.0 |
| **HsTrm112** | Identity (%) | 20.0 | 34.5 | 33.9 | - |
|  | RMSD (Å) | 1.8 | 1.5 | 1.5 | - |
|  | Aligned Cα | 95 | 116 | 115 | - |
|  | Z-score | 11.5 | 17.3 | 16.3 | - |

Supplementary Figure Legends

Supplementary Figure S1. A representative composite simulated annealing omit map (*2Fo–Fc*, contoured at 1.0 σ level) showing the region of the extra visible N-terminal L1 loop of N6amt1 in the Se-Met derivative complex structure. Residues of the L1 loop are shown with a green ball-and-stick model.

Supplementary Figure S2. Structural comparisons of HsTrm112 with yeast and protozoan Trm112 in other MTase-Trm112 complexes. (a) Structural comparisons of HsTrm112 with EcTrm112 (left panel, PDB code 3Q87), YlTrm112 (middle panel, PDB code 5CM2), and ScTrm112 (right panel, PDB code 4QTT), respectively. The color schemes of Trm112 in different protein complexes are shown above. (b) Detailed interaction networks of the zinc-binding motif of EcTrm112 (left panel), YlTrm112 (middle panel), and ScTrm112 (right panel). The residues involved in the interaction network are shown with ball-and-stick models, and the Zn^2+^ ion is shown with a grey sphere. The hydrogen bonds are indicated with black dotted lines.

Supplementary Figure S3. Sequence alignments of (a) Trm112 and (b) N6amt1 from different species. The sequence numbers and secondary structures of HsTrm112 and HsN6amt1 are placed on the tops of the alignments. The abbreviations for the species are as follows: Hs, *Homo sapiens*; Mm, *Mus musculus*; Rn, *Rattus norvegicus*; Gg, *Gallus gallus*; Bt, *Bos taurus*; Dr, *Danio rerio*; Dm, *Drosophila melanogaster*; Xl, *Xenopus laevis*; and Sc, *Saccharomyces cerevisiae*. Strictly conserved residues are highlighted in shaded red boxes and conserved residues in open red boxes. The structure elements constituting the accessory structure elements beyond the MTase domain of N6amt1 are highlighted in blue boxes. The key residues of HsN6amt1 involved in the cofactor binding are marked with green dots. The key residues involved in the interaction between HsN6amt1 and HsTrm112 are marked with yellow triangles. The key residues constituting the “S(R/K)CS motif” of HsTrm112 are marked with blue asterisks.

Supplementary Figure S4. Structural comparisons of human N6amt1-Trm112 complex with *E. coli* HemK and other MTase-Trm112 complexes. (a) Structural comparisons of HsN6amt1-Trm112 with EcMtq2-Trm112 (PDB code 3Q87), EcHemK-RF1 (PDB code 2B3T), YlTrm9-Trm112 (PDB code 5CM2), and ScBud23-Trm112 (PDB code 4QTT). The color schemes of different proteins or protein complexes are shown above. (b) Electrostatic potential surfaces of EcHemK in complex with RF1, and EcMtq2, YlTrm9, and ScBud23 in different MTase-Trm112 complexes. RF1 and Trm112 in different complexes are shown with ribbon models. (c) Structural comparison of the MTase-Trm112 interface in different complexes. Several hydrophilic interactions between N6amt1 and Trm112 are also found in the YlTrm9-Trm112 and ScBud23-Trm112 complexes. The residues involved in the interactions are shown with ball-and-stick models and the residues of Trm112 are labeled in italics. Hydrogen-bonding interactions are indicated with black dotted lines. (d) Structure of the active site of EcHemK. The glutamine residue of RF1 is inserted into the active site of HemK and stabilized by the NPPY motif. (e) Structural comparison of the active site of N6amt1-Trm112 in complex with an H4K12me1 peptide (PDB code 6H1E) and the active site of N6amt1-Trm112 docked with a glutamine residue based on the superposition of *E. coli* HemK2-RF1 complex onto N6amt1-Trm112. The residues of N6amt1, the bound SAH, and the monomethylated lysine of the H4K12me1 peptide in the N6amt1-Trm112-H4K12me1 complex are shown with ball-and-stick models and colored in violet, gray, and wheat, respectively, and the residues of N6amt1, the bound SAM, and the docked glutamine residue in the N6amt1-Trm112 complex are colored in salmon, yellow, and green, respectively.

Supplementary Figure S1


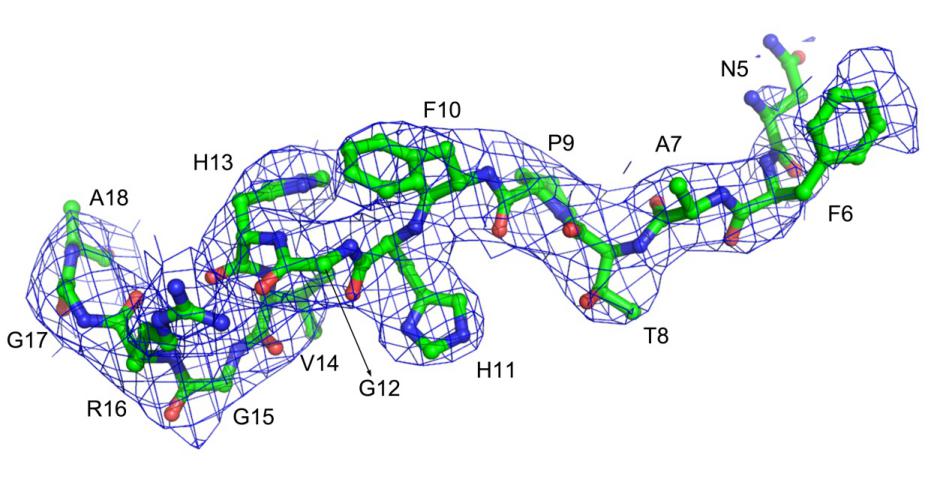


Supplementary Figure S2

a


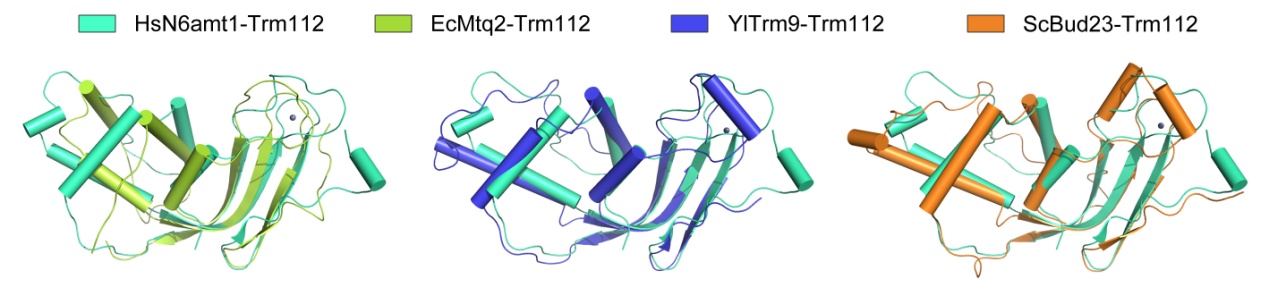


b


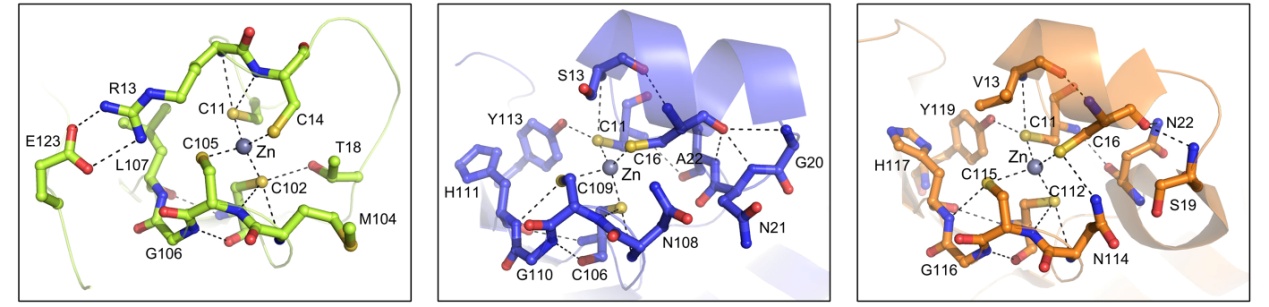


Supplementary Figure S3

a


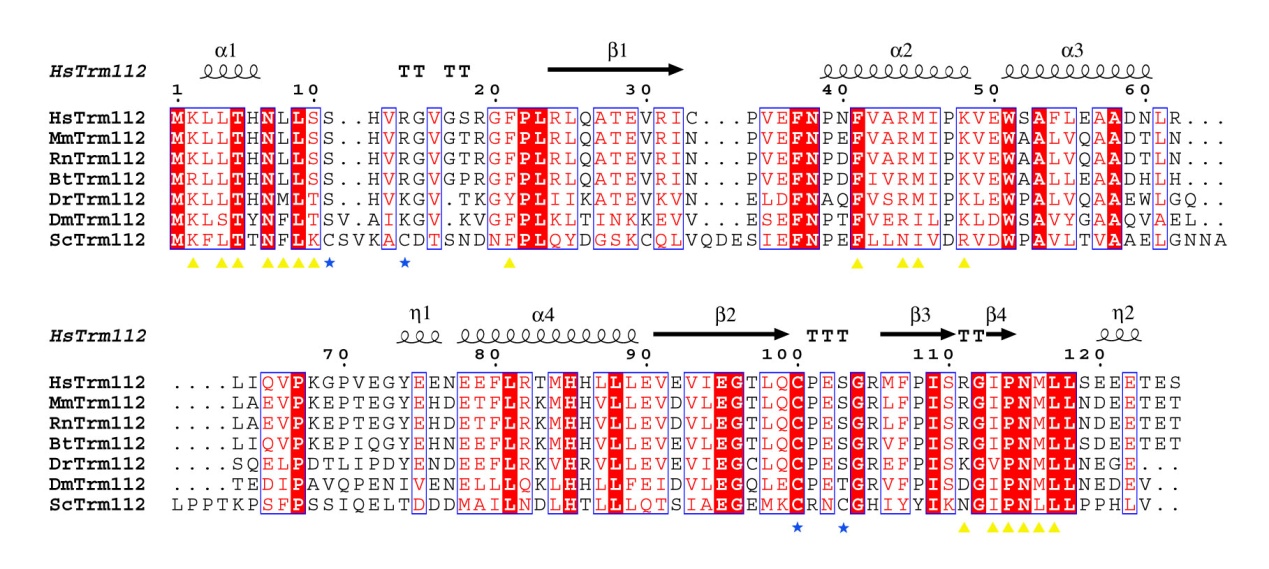


b


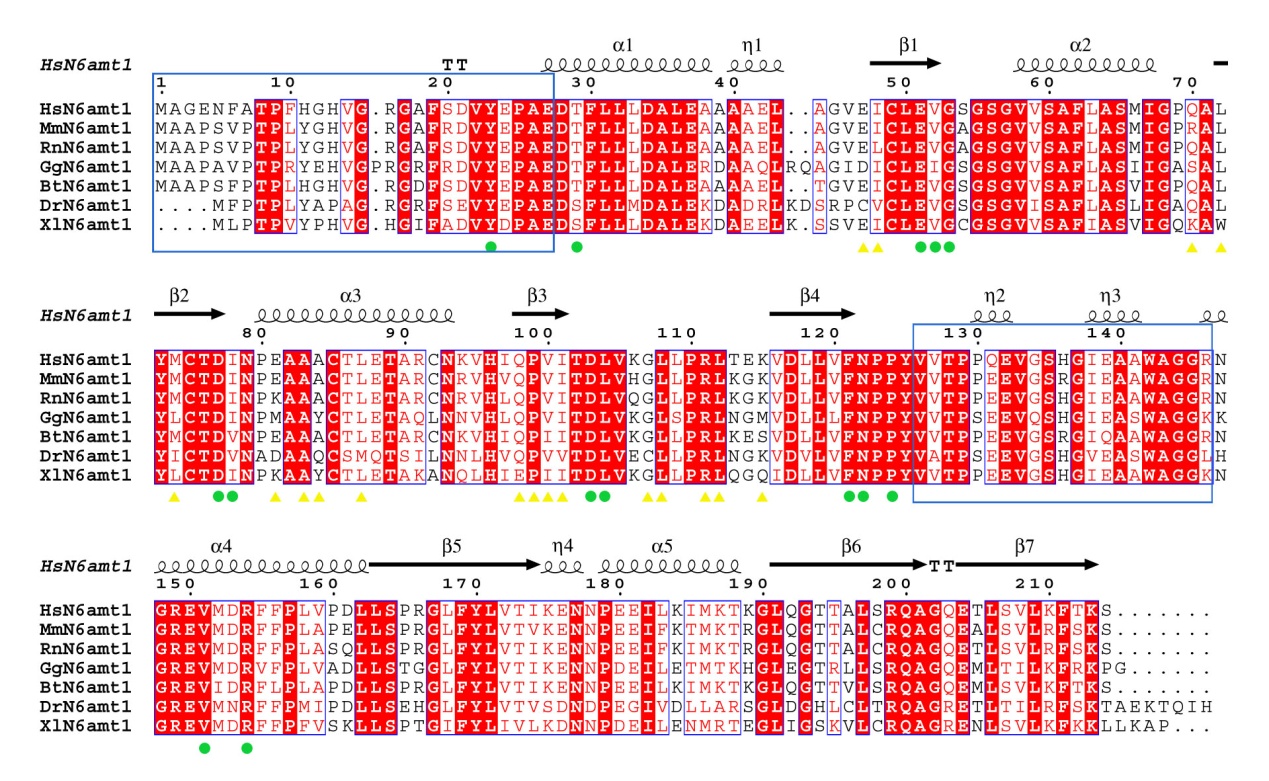


Supplementary Figure S4

a


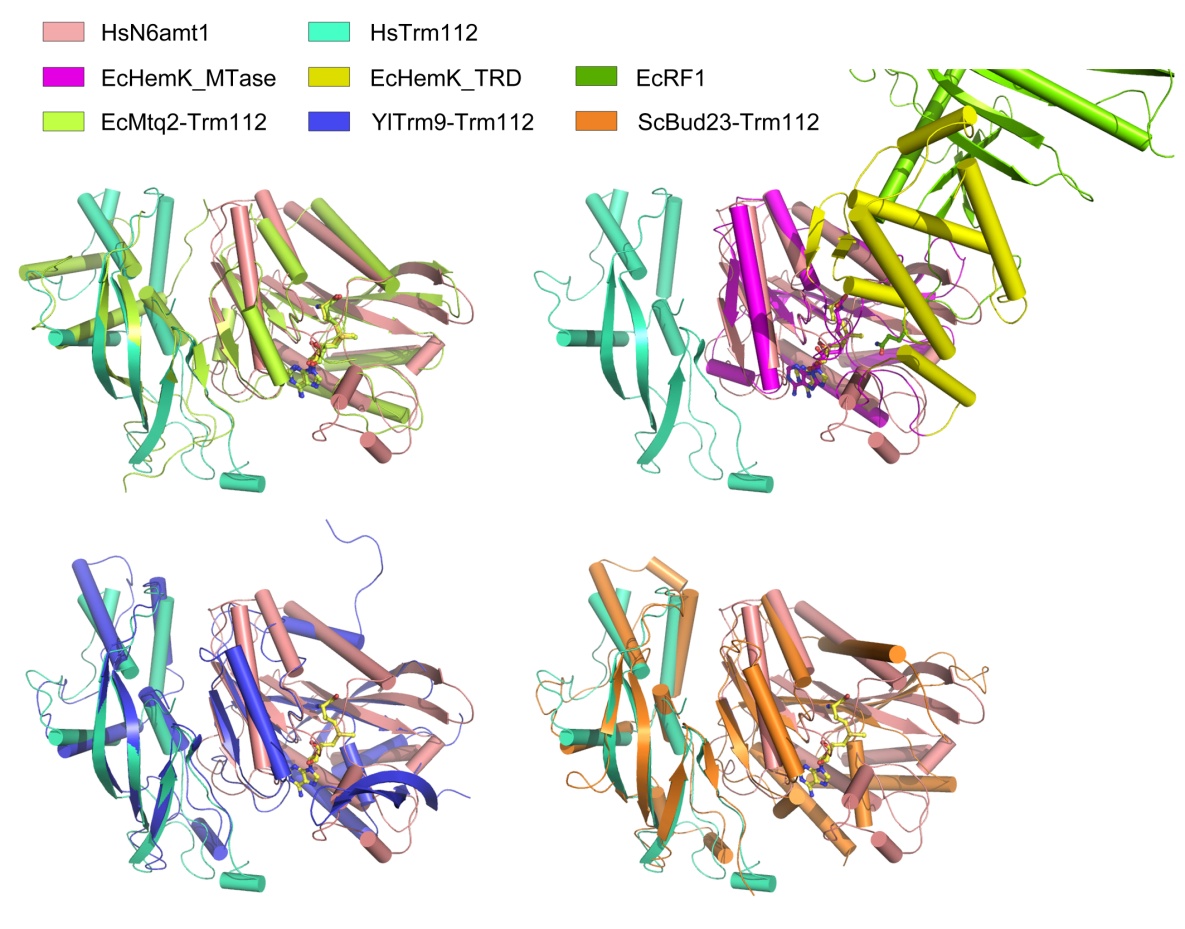


b


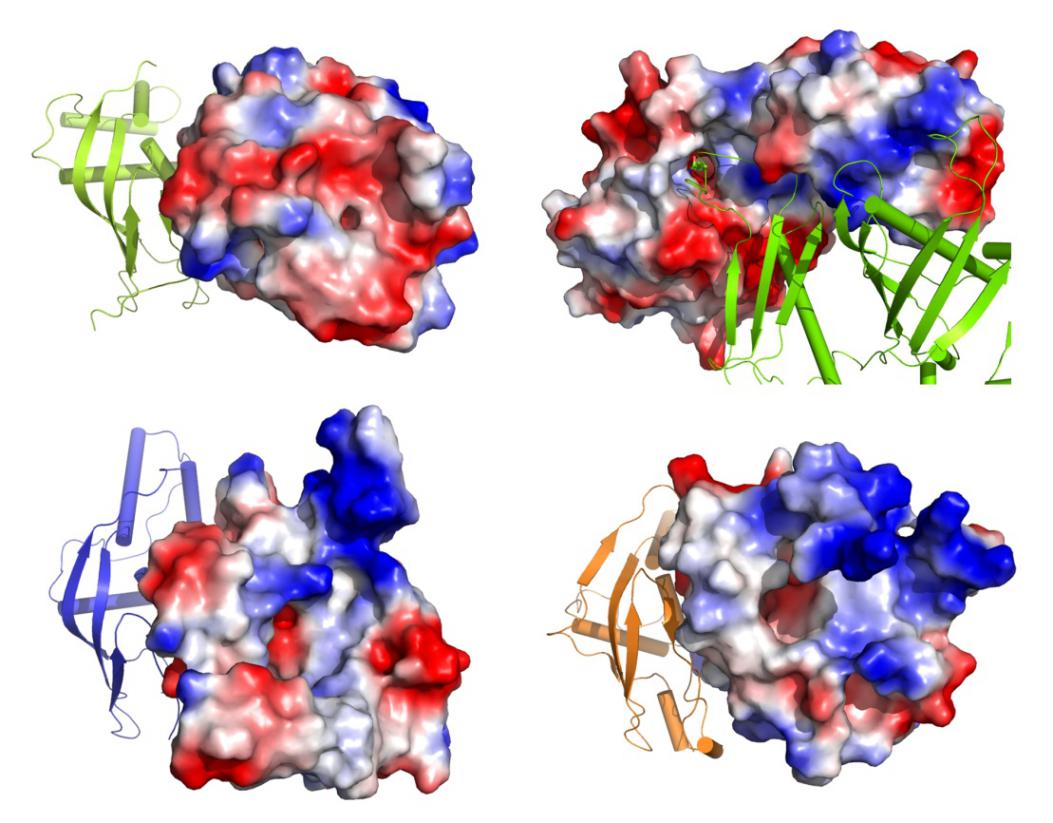


**c**


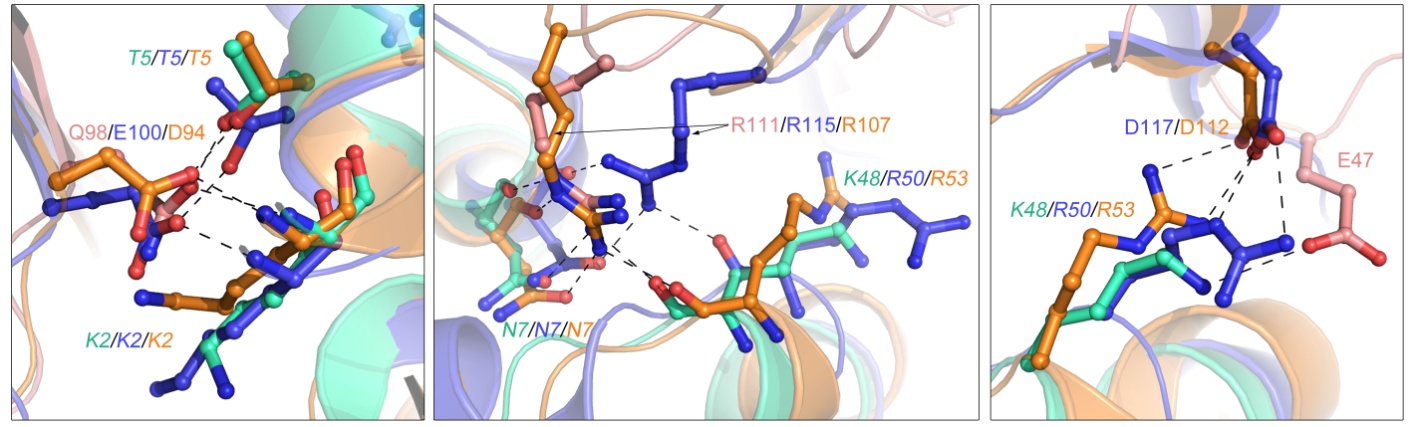


**d e**

**
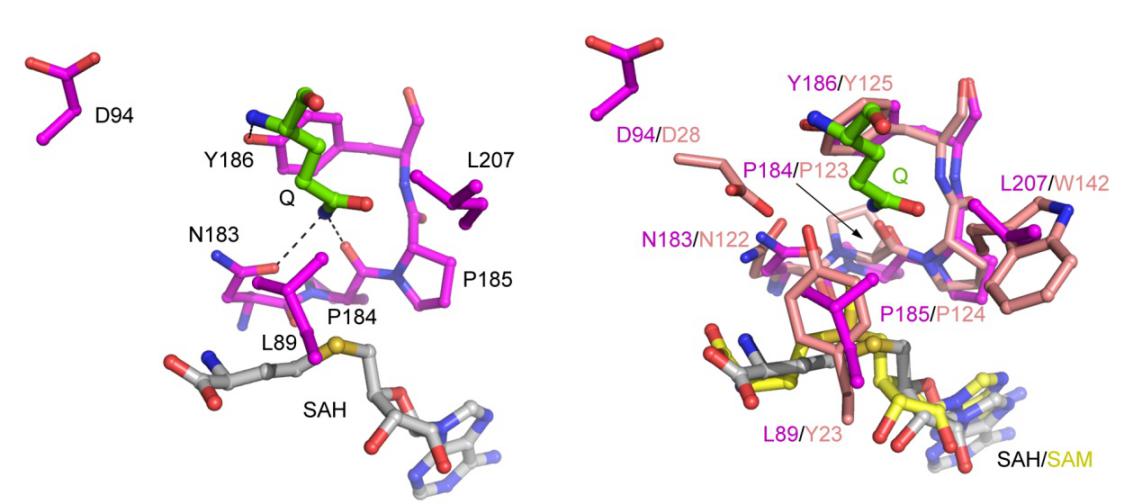

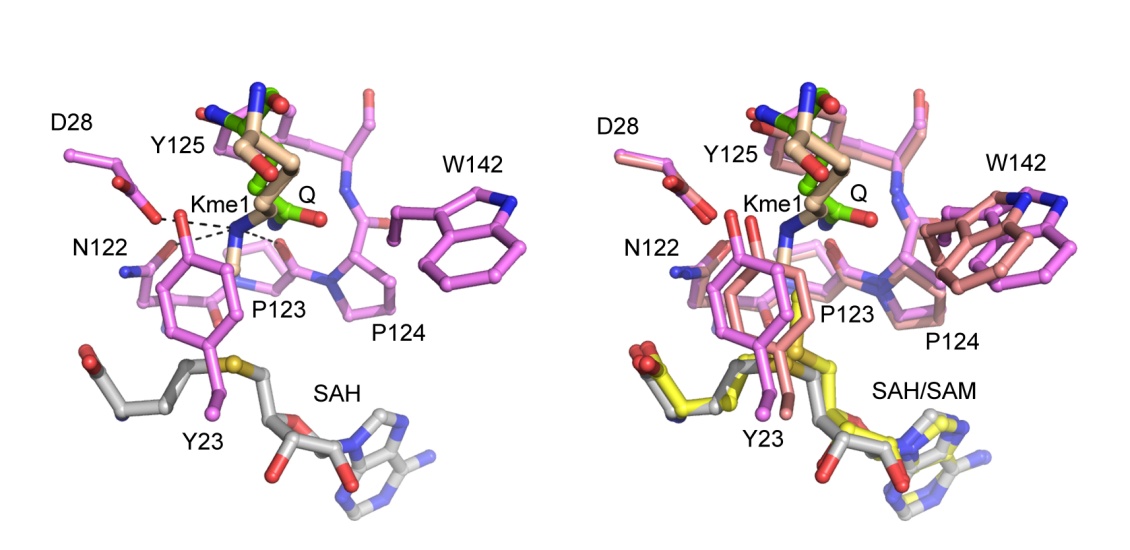
**
